# Supplementary material for: CRISPR/Cas9-mediated ApoE-/- and LDLR-/- double gene knockout in pigs elevates serum LDL-C and TC levels
Source: Oncotarget. 2017 Apr 17;8(23):37751–60. doi: 10.18632/oncotarget.17154 (PMC5514946; doi:10.18632/oncotarget.17154)
Supplement: Supplementary file 1 [file oncotarget-08-37751-s001.pdf]

CRISPR/Cas9-mediated *ApoE*$^{-/-}$ and *LDLR*$^{-/-}$ double gene knockout in pigs elevates serum LDL-C and TC levels

SUPPLEMENTARY FIGURES AND TABLES

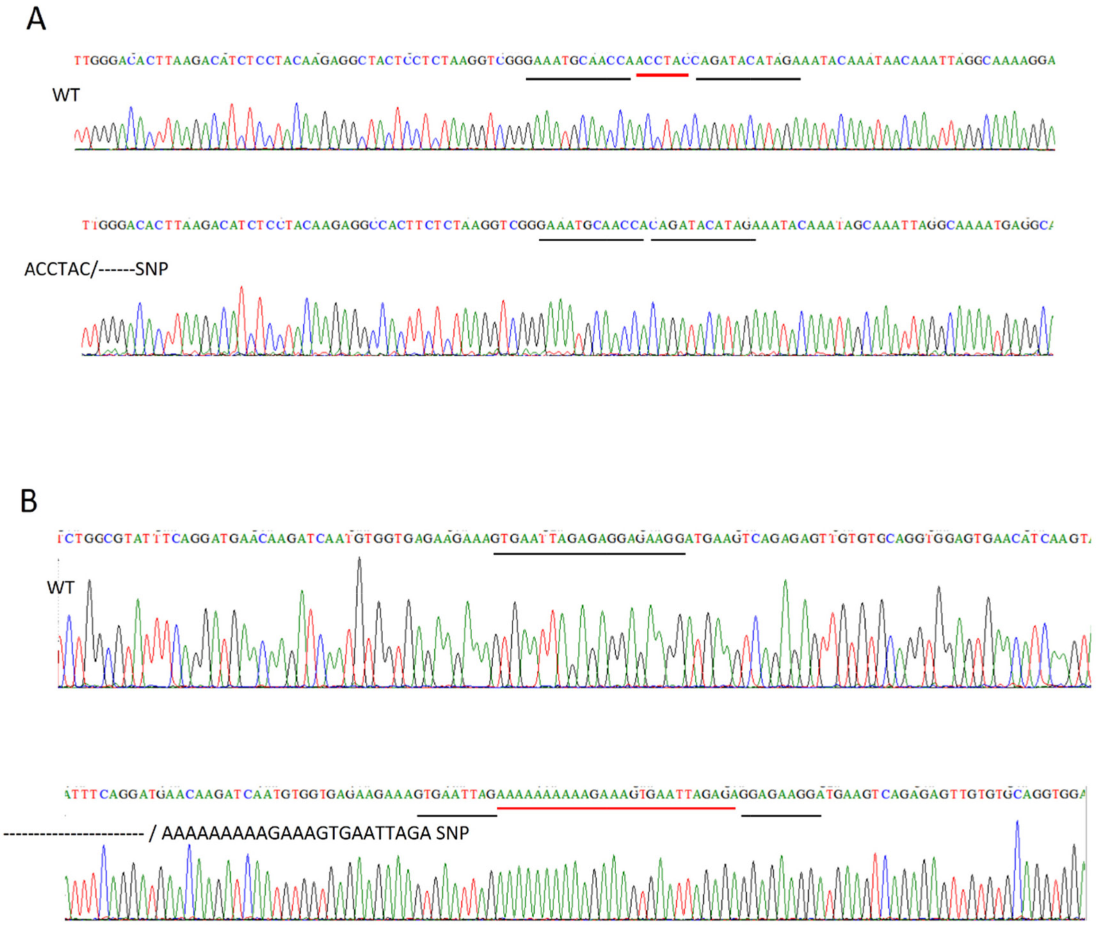

Supplementary Figure 1: SNPs around the LDLR-OT5 and LDLR-OT6.

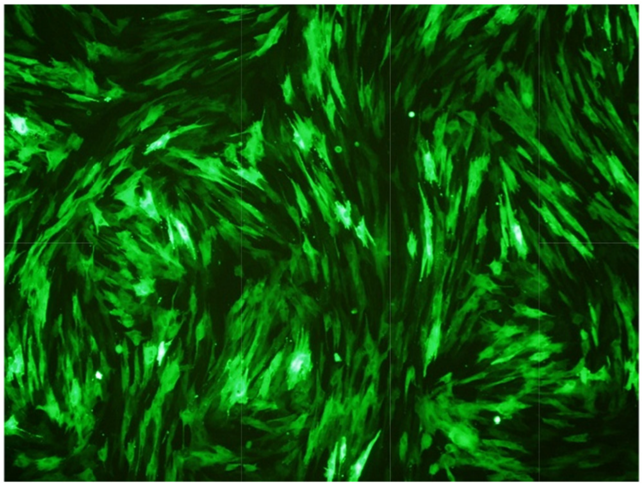

Supplementary Figure 2: Transfection efficiency evaluated with EGFP plasmid.

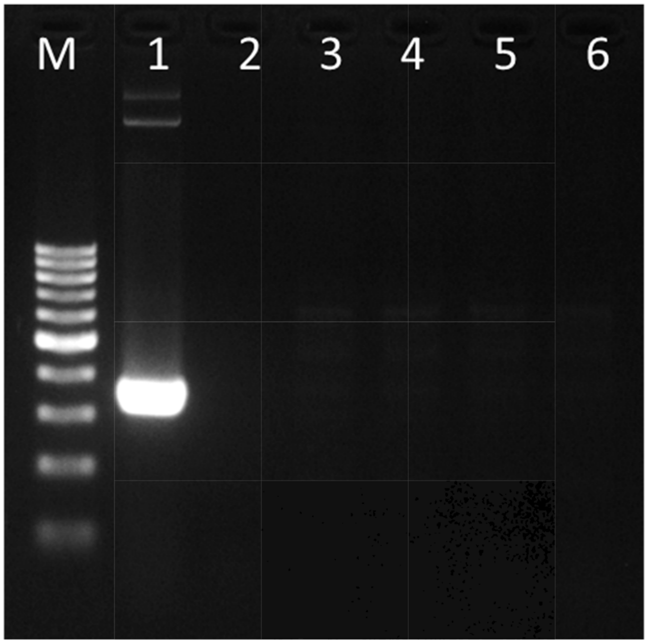

Supplementary Figure 3: Detection of genomic plasmid integration. Lane 1, positive control of plasmid, lane 2, negative control of water, lane 3-6, four pigs of F1 generation.

Supplementary Table 1: TA cloning assay of gRNAs

	Total NO.	NO. of mutation	Mutation rate
gRNA 1	22	19	86.3%
gRNA 2	16	2	12.5%
gRNA 3	30	8	26.7%
gRNA 4	31	3	9.7%

Supplementary Table 2: Primer information of *ApoE* and *LDLR* gene

Primer name	Forward primer(5'—3')	Reverse primer(5'—3')	size (bp)
ApoE	GCAGGGCGTGAGCATTAGAT	AGGACGGCAAGACTGACCCA	698
LDLR1	ACAGGGAGTATGGTCACTTGC	AAATACCTTCTCCGCCCACA	304
LDLR2	CCTCCACGATGTTGTTGGTT	GACCAGTTGGTAAGGGCTAT	850

Supplementary Table 3: primers of off-targets

See Supplementary File 1
