# Supplementary material for: CRISPR/Cas9-mediated ApoE-/- and LDLR-/- double gene knockout in pigs elevates serum LDL-C and TC levels
Source: Oncotarget. 2017 Apr 17;8(23):37751–60. doi: 10.18632/oncotarget.17154 (PMC5514946; doi:10.18632/oncotarget.17154)
Supplement: Supplementary file 2 [file oncotarget-08-37751-s002.docx]

CRISPR/Cas9-mediated *ApoE*^-/-^ and *LDLR*^-/-^ double gene knockout in pigs elevates serum LDL-C and TC levels

Lei Huang^1,3^, Zaidong Hua^2^, Hongwei Xiao^2^, Ying Cheng^1^, Kui Xu^1^, Qian Gao^1^, Ying Xia^1^, Yang Liu^1^, Xue Zhang^1^, Xinming Zheng^2^, Yulian Mu^1^, Kui Li^1^

^1^State Key Laboratory of Animal Nutrition, Institute of Animal Sciences, Chinese Academy of Agricultural Sciences, Beijing 100193, China

^2^Hubei Key Laboratory of Animal Embryo Engineering and Molecular Breeding, Institute of Animal Science and Veterinary Medicine, Hubei Academy of Agricultural Science, Wuhan 430064, China

^3^Animal Functional Genomics Group, Agricultural Genomes Institute at Shenzhen, Chinese Academy of Agricultural Sciences, Shenzhen 518120, China

**Correspondence:** Yulian Mu, email: mouyulian@caas.cn

Keywords:

cardiovascular disease, dyslipidemia, animal model, multiple-gene knockout, gene editing

Received: September 06, 2016 Accepted: March 28, 2017 Published: April 17, 2017

ABSTRACT

The traditional method to establish a cardiovascular disease model induced by high fat and high cholesterol diets is time consuming and laborious and may not be appropriate in all circumstances. A suitable pig model to study metabolic disorders and subsequent atherosclerosis is not currently available. For this purpose, we applied the CRISPR/Cas9 system to Bama minipigs, targeting apolipoprotein E *(ApoE)* and low density lipoprotein receptor *(LDLR)* gene simultaneously. Six biallelic knockout pigs of these two genes were obtained successfully in a single step. No off-target incidents or mosaic mutations were detected by an unbiased analysis. Serum biochemical analyses of gene-modified piglets showed that the levels of low density lipoprotein choleserol (LDL-C), total cholesterol (TC) and apolipoprotein B (APOB) were elevated significantly. This model should prove valuable for the study of human cardiovascular disease and related translational research.

INTRODUCTION

Atherosclerosis is primarily caused by the imbalance of blood lipids, particularly cholesterol-containing low density lipoprotein (LDL) particles [1]. *ApoE* and *LDLR* gene mutations play a large role in the disease progression. ApoE protein has a great effect on cholesterol metabolism in the liver and is an important component of very low density lipoprotein (VLDL) and chylomicrons. The functional deficiency of ApoE protein contributes to the risk of familial dysbetalipoproteinemia or type III hyperlipoproteinemia (HLP III) [2]. LDLR protein, when located on the surface of hepatocytes, plays a critical role in low density lipoprotein cholesterol (LDL-C) clearance in liver. Loss-of-function mutations in this gene result in the accumulation of LDL-C in circulation, leading to familial hypercholesterolemia (FH) [3]. *ApoE*^-/-^/*LDLR*^-/-^ mice have been broadly used in atherosclerosis research; however, lipoprotein profiles and metabolism in mice are different from humans and pigs [4]. For example, most of the cholesterol of mice was transported by high density lipoprotein (HDL). However, in humans and pigs it was mainly through the LDL. Furthermore, pig models could reproduce many important atherosclerosis features, such as palque rupture and thrombosis. However, these features were hardly ever observed in mouse models [5].

Pigs are widely used in biomedical and human diseases research [6]. They share many similarities to humans with regard to nutrition, anatomy, physiology, pathology and the cardiovascular system [7]. For example, their heart size, blood supply, function of coronary system and feature of aorta are comparable to humans [8]. Thus the pig is an important model of cardiovascular disease research. However, pig atherosclerosis process is quite slow on normal physiological condition. So the diet inducing or gene modification is needed in this model. During the last five years, numerous genetically modified pigs have been produced using gene targeting approaches to model human metabolic diseases, neurodevelopmental disorders, immunodeficiency and muscle development [6, 7, 9–14]. Gene edited pig models of dyslipidemia or lipid metabolism disorders provide large amounts of material to bridge the gap between preclinical evaluations in humans and rodents [15]. However, few pig models related to atherosclerosis have been generated using gene editing methods.

CRISPR/Cas9 is a newly emerging versatile genome engineering tool, which is composed of a single guide RNA (gRNA) and the Cas9 enzyme for genome cutting [16]. This system exhibits the merits of convenience, versatility and high efficiency for targeted genome editing compared with ZFNs and TALENs [17]. In the past two years, the CRISPR/Cas9 system has been successfully used to introduce zygotes to generate gene-edited pigs [9, 11, 14, 18–20]. However, CRISPR/Cas9-mediated cleavage would occur at the early embryonic development stage multiple times, which might result in chimeric piglets with multiple genotypes [18]. Therefore, the founder piglets exhibit more than two mutations in different tissues, including the gonads. Thus, one or two rounds of additional breeding to obtain stable genetic offspring are required [12]. This procedure is time-consuming and costly in pigs, which have long gestation cycles. The CRISPR/Cas9 system has been used to target two genes in pigs for establishing a neurodegenerative disease model [12]. As such, we aimed to target multiple genes related to arthrosclerosis in pigs simultaneously. Hence, we utilized the CRISPR/Cas9 system to mediate gene editing in porcine embryonic fibroblasts (PEFs). These somatic cells, which carry stable and identical mutations, were then used as nuclear donors to produce the knockout pigs described in this study.

Here, we aimed to generate *ApoE*^-/-^/*LDLR*^-/-^ double knockout pigs with biallelic mutations via the CRISPR/Cas9 system and SCNT approach. These genetically modified pigs will provide an effective model for atherosclerosis studies.

RESULTS

CRISPR/Cas9 construction and efficiency tests of the designed gRNAs

To verify whether single nucleotide polymorphisms (SNPs) existed in these two loci, the nuclear acids of exon2 for *ApoE* and *LDLR* genes were amplified and sequenced for gRNA design (Figure 1). The targeted regions of gRNAs for the *ApoE* and *LDLR* genes are shown in Figure 1A and 1B. After transfection, the sequencing chromatography of the two targeted genes showed overlapped peaks around the PAM sequence (Figure 1), indicating that only two gRNAs could induce indels in the target region and with different mutagenesis efficacies. However, as the bacterial TA colony analysis showed, as summarized in Supplementary Table 1, all four gRNAs could induce indels in the target gene and the mutation efficiencies ranged from 9.7% to 26.7% for the *LDLR* gene and from 12.5% to 86.3% for the *ApoE* gene. Consistent with our sub-clone analysis, the T7EN1 digestion analysis showed gRNA1 and gRNA3 had high rates of mutation genesis (Figure 1C). However, for gRNA2 and gRNA4, the digestion failed, suggesting the rates (12.5% and 9.7%) were under the detection capability of T7EN1 enzyme.

Figure 1

Design and CRISPR/Cas9 assay.

**(A, B)** Schematic and sequencing chromatography of genomic DNA of the CRISPR/Cas9-targeting sites in pig *APOE* **(A)** and *LDLR* **(B)** genes. Exons are shown as boxes. The black arrow indicates the direction of transcription. The gRNA-targeting sequence is labeled in red, and the protospacer-adjacent motif (PAM) sequence is labeled in green. The different gRNAs are named as gRNA 1-4, respectively. Sequencing chromatography of genomic DNA of *ApoE* **(A)** and *LDLR* **(B)** genes showd a double curve after the guided double strand break induced by the CRISPR/Cas9. **(C)** Agarose gel electrophoresis showing the PCR product of the target region derived from PEF cell lines digested with T7EN1 restriction enzymes. WT:wild type; g1:gRNA1; g3:gRNA3.

PEF cell transfection and gRNA-induced site-specific indels of pig *ApoE* and *LDLR* genes

To target *ApoE* and *LDLR* genes simultaneously and to investigate whether the colony formation rate had a positive correlation with the gRNA mutation genesis, cas9 and gRNA1 (high 86.3%) were cotransfected with gRNA3 (moderate 26.7%) or gRNA4 (low 9.7%). After about 10 d in culture, the colonies were harvested, PCR was performed on each colony for identification, and Sanger sequencing was performed for each gene. For the *ApoE* gene, among all 62 colonies checked, the deletion range was from 1 bp to 247 bp, and the insertions were 1-2 bp (Figure 2). The monoallelic and biallelic mutants were 26% and 55% of the total, respectively. For the *LDLR* gene, 26 gRNA3 targeted colonies were checked; the deletion and insertions were 4-132 bp and 1-83 bp, respectively, and the monoallelic and biallelic mutant rates were 19% and 31%, respectively. The 22 gRNA4 targeted colonies showed 32% and 14% monoallelic and biallelic mutant rates, respectively (Table 1). These data indicated a positive correlation but a relatively narrowly gap of mutagenesis efficacies between gRNAs with high, moderate and low levels (Table 1). We had obtained 8 biallelic mutants for *LDLR* gRNA3 and 3 for *LDLR* gRNA4. Fortunately, all the 11 cell colonies with *LDLR* biallelic mutants also had biallelic mutants for *ApoE* (Table 1 and Figure 3).

Figure 2

Mutations of APOE and LDLR genesis by CRISPR/Cas9.

Detailed mutations of the modified alleles detected in PEF colonies. gRNA binding sites of APOE and LDLR gene are underlined; “Δ”: deletion; “+”: insertion; WT: wild-type allele.

Table 1

The efficacy of CRISPR/Cas9-mediated gene targeting in PEFs

| **Target genes** | | **Mutant alleles (Mutated colony/tested colonies)** | | |
| --- | --- | --- | --- | --- |
|  |  | **Monoallelic mutant (%)** | **Biallelic mutant (%)** | **Total mutant (%)** |
| *ApoE* |  | 16/62 (26%) | 34/62 (55%) | 50/62 (81%) |
| *LDLR* | g3 | 5/26 (19%) | 8/26 (31%) | 13/26 (50%) |
|  | g4 | 7/22 (32%) | 3/22 (14%) | 10/22 (45%) |
| *ApoE* | *LDLR*-g3 | - | 8* | - |
|  | *LDLR*-g4 | - | 3* | - |

Notes: * Both the 8 and 3 colonies were also had biallelic mutants for *ApoE*. g3:gRNA3; g4:gRNA4.

Figure 3

*ApoE/LDLR* double-mutant cell colonies for SCNT.

The chromatogram for a partial genomic sequence of *ApoE/LDLR* double mutant PEF colonies. Sequencing was used to verify insertions or deletions produced by the non-homologous end joining repair pathway after the double strand break induced by the CRISPR/Cas9. gRNA binding sites are underlined; “Δ”: deletion; “+”: insertion; WT: wild-type allele of *ApoE* and *LDLR* gene, respectively.

Production of *ApoE*/*LDLR* knockout pigs by SCNT and genotype assay

*ApoE*/*LDLR* double-mutant cell colonies (5# and 7#) (Figure 3) were selected as SCNT donors, and nearly 180-300 reconstructed embryos were transferred to surrogates. Each cell colonony was used in four surrogates. One of these eight sows which was transferred with 7# cell colony generated six piglets. These offsprings were live born (Figure 4A) and genotyped. Mutations corresponding to the 7# donor colony were found at the target loci for all 6 founder piglets (Figure 4A).

Figure 4

*ApoE/LDLR* knockout pigs and serum biochemical status.

**(A)** Photographs and genotypes of the six *ApoE*^-/-^/*LDLR*^-/-^ founder pigs. **(B)** Serum TC, TG, LDL-C and HDL-C in male founders and WT males. The data comprise fasting serum measurements at two-months and twelve-months of age in founders and age-matched WT pigs. **(C)** Serum APOB and ApoE levels at two-months and twelve-months of age. The data comprise fasting serum measurements at two-months and twelve-months of age in founders and age-matched WT pigs. * P<0.05, ** P<0.01.

Off-target analysis of gene-targeted pigs

A total of 28 (12 for *ApoE* and 16 for *LDLR*) potential off-target sites were successfully amplified and subjected to Sanger sequencing via PCR. Two sites (*LDLR*-OT5 and *LDLR*-OT6) in these 28 off-target sites (OTs) were detected with overlapped peaks around the OTs. To further verify if off-target cleavage events occurred, the PCR products from *LDLR*-OT5 and *LDLR*-OT6 were subjected to TA-clone and subsequent Sanger sequencing. SNPs were observed in these OTs (Supplementary Figure 1A): *LDLR*-OT5 contained a ACCTAC/------ SNP (HGVS name:1:g.57898317_57898322delACCTAC), and *LDLR*-OT6 contained a -----------------------/AAAAAAAAAGAAAGTGAATTAGA SNP 58bp upstream from this OT (Supplementary Figure 1B). For the remaining 26 OTs, no CRISPR/Cas9-induced mutations were detected in any of the six target gene–disrupted pigs.

Serum biochemical analyses

To assess the potential effects of *ApoE*/*LDLR* gene mutation on serum lipids, plasma samples collected from the knockout pigs and control pigs were analyzed. Plasma high density lipoprotein cholesterol (HDL-C), LDL-C, TC and triglyceride (TG) levels were measured in two-month-old *ApoE*^-/-^/*LDLR*^-/-^ and *ApoE*^+/+^/*LDLR*^+/+^ piglets (Figure 4B, 4C). As expected, TC and TG levels were significantly higher in *ApoE*^-/-^/*LDLR*^-/-^ pigs than in wild-type pigs (TC, 3.05 ± 0.12 mM, mean ± SEM VS 1.94 ± 0.16 mM, P=0.0002; TG, 0.77 ± 0.08 mM VS 0.35 ± 0.06 mM, (P=0.0015, t-test). A similar elevation is observed in LDL-C and APOB, which is the main apolipoprotein of LDL (LDL-C, 1.96 ± 0.06 mM, mean ± SEM VS 1.39 ± 0.13 mM, P=0.0030; APOB, 186.21 ± 6.15 μg/mL mean ± SEM VS 144.37 ± 12.56 μg/mL) (P=0.0115, t-test). Surprisingly, HDL-C was significantly elevated in *APOE*^-/-^/*LDLR*^-/-^ pigs (1.15 ± 0.06 mM, mean ± SEM VS 0.83 ± 0.06 mM, P=0.0039, t-test). This result was corresponded with our another artherosclerosis pig model established by means of high cholesterol and fat food inducing (data not published). Meanwhile, the TC, LDL-C and HDL-C was also significantly elevated (TC, P=0.0281; LDL-C, P=0.0276; HDL-C, P=0.0029) in knockout pigs of twelve month old twelve-month-old (Figure 4B).

DISCUSSION

As the “third generation” of gene editing systems, distinct from ZFNs and TALENs, CRISPR/Cas9 is easily applicable and low cost. Thus, it provides a robust tool for deleting target genes and creating genetically modified large livestock animals such as pigs. Considering the random genotypes and chimeric piglet generated from a one-cell stage co-injection of Cas9 mRNA and gRNA, in this study, we used the CRISPR/Cas9 system combined SCNT to generate *ApoE*/*LDLR* gene modified pigs and obtained double gene knockout animals with a single round of the procedure. The circular plasmid of CRISPR/Cas9 system could not be integrated into the genome; we just needed to enrich transfected cells with transient expression of puromycin for about 3 to 5 days. And then another 7 days of culture would generate cell colony with designed mutation. To confirm whether puromycin marker was integrated into the genome of pigs, we attempted to do the detection of genomic plamid integration. As showed in Supplementary Figure 3, four pigs of F1 generation did not have any puromycin marker integration. Thus, we are not concerned about the constitutive expression of CRISPR/Cas9, which would increase the frequencies of off-target mutations [7]. Additionally, the antibiotic-mediated cell enrichment for several days could increase screening efficiency without affecting cell viability. Obviously, we found a high probability biallelic knockout, consistent with the results of previous studies [7, 12, 18]. Hence, the CRISPR/Cas9 system demonstrated an enhanced ability to introduce a biallelic mutant on homologous chromosomes, which permitted us to edit multiple genes simultaneously.

Because of the metabolic and immune similarities between pigs and humans, the establishment of swine models is important for the study of disease-related genes. ApoE is a polymorphic protein, and there is a significant association between molecular polymorphisms and several biological processes, including Alzheimer's disease, cognitive function, immunoregulation, cerebrovascular and cardiovascular pathologies and possibly infectious diseases. The polymorphisms of the *LDLR* gene are the most frequent genetic cause of FH in humans and are linked to coronary heart disease in humans, mice, and pigs. By introducing *ApoE*/*LDLR* mutations into the pig genome, we found that serum LDL-C was elevated by approximately 41% from WT at an early age. Furthermore, APOB, the main apolipoprotein component on LDL, increased approximately 29% in genetically modified pigs. We also observed that there was nearly a 57% elevation of serum total cholesterol and a 120% elevation of serum TG. Considering all the serum biochemical analyses were processed in two-month-old weanling piglets, it could reflect the real physiological state of the juvenile piglets and totally eliminate the effects of lipids in sow milk. We also confirmed that the increased TC and LDL-C in mutant piglet serum could be persisted in long-term observation. The serum of twelve-month-old knockout and WT pigs were collected for biochemical analyses. As we expected, TC and LDL-C was still increased significantly in twelve-month-old knockout pigs (Figure 4B). One can speculate that the knockout of *ApoE* and *LDLR* genes in pigs did increase their susceptibility to cardiovascular diseases at an early age and could recreate the same situation as human cardio-metabolic disease. Several type of *LDLR* knockout pigs have been used for atherosclerosis research [3, 21, 22]. Davis et al [3] and Li Y et al [21] generated *LDLR* knockout pigs through disrupting exon 4. They used conventional homologous recombination to insert resistance genes into such region to achieve the purpose of destroying *LDLR* gene. These models have significant dyslipidemia phenotypes, such as a significant rise in TC and LDL-C levels. In this study, we used CRISPR/Cas9 to edit exon 2 of of *LDLR* gene, and obtained a similar pig model of dyslipidemia.

Off-targets are of great importance to be considered when using the CRISPR/Cas9 genome engineering system. Base pairing between the gRNAs and the target sequence will affect the off-target mutations rates. A short seed sequence (usually the first 10-12 nucleotides adjacent to PAM) was found to be generally more important than the rest of the guide region. Additionally, several groups independently reported that more than five mismatches in total and more than two mismatches in the seed sequence will most likely prevent CRISPR/Cas9-mediated DSB induction. Notably, we administered a relatively stringent rule, such as the 0-1 mismatch of the 12 nt seed sequence and the 1-5 mismatch of the total, yet we failed to identify any off-target incidents in all of the 28 off-targets detected. Furthermore, CRISPR/Cas9-mediated gene disruption demonstrated high fidelity for each target sequence among all six founders because no off-target mutations were detected at any of the predicted 28 off-target sites. Even for these three sites, the most potential off-target sites, *ApoE*-OT2 (with 1 nt mismatch within the 12 nt seed sequence and only 2 nt mismatch total), *ApoE*-OT3 and *LDLR*-OT1 (with an exact match of a 12 nt seed sequence and only a 3 mismatch total), there were no off-target mutagenesis detected. There has been speculation that particular gene-modified cell colonies may exhibit poor competency to develop to term because of off-target side effects. This probably explains why there was no off-target mutagenesis detectable. Nonetheless, this result is based on a limited locus; more sensitive and unbiased detection of genome-wide mutations will be needed to determine if CRISPR/Cas9 induced off-target cutting. Moreover, the consistency of the genetic background of the six founders also suggests that no CRISPR/Cas9-mediated DNA cleavage occurred at the one-cell stage or the later embryonic stage. These results testify to the effectiveness of puromycin enrichment of resistant cells after circular plasmid transfection.

In conclusion, herein, we presented the successful generation of genetically modified pigs targeting the *ApoE* and *LDLR* genes simultaneously via the CRISPR/Cas9 approach. The gene-modified swine showed an abnormal lipid metabolism related to atherosclerosis. This will aid in determining the gene function in pigs and in establishing valuable animal models for the study of human cardiovascular disease.

MATERIALS AND METHODS

Ethics statement

The pigs used in this study were maintained in the National Germplasm Resources Center of Laboratory Miniature Pig (Beijing, China). All pig experiments were conducted in accordance to the guidelines provided by the Committee on the Ethics of Animal Experiments of the Institute of Animal Science of Chinese Academy of Agriculture Sciences (IAS of CAAS) (Beijing, China). The protocols were approved by the IAS of CAAS.

CRISPR/Cas9 construction

U6 promoter-driven gRNA cloning vector pGL3-U6-gRNA-PGK-puromycin (51133) and Cas9 expressing plasmid (44758) were purchased from Addgene (Cambridge, USA). Four gRNAs targeting exon 2 of pig *ApoE* or *LDLR* were designed following the protocol described previously [19]. Briefly, 2 gRNAs for *ApoE* and *LDLR* genes were selected based on the original Cas9 approach G-18(19)N-GG. Each pair of complementary DNA oligos were synthesized, subsequently dephosphorylated, and then annealed to double-strand fragments with BbsI-compatible overhangs. All constructs were subjected to Sanger sequencing (Taihe Biotechnology, Beijing, China) and then electroporated into PEF cells individually. PCR products flanking the target site were sequenced and then analyzed by T7EN1 and TA clones for identification of the mutagenic efficacy.

Targeting efficiency of gRNAs

PEF cell lines used in this study were collected from the Chinese Bama miniature pig as described previously by our group [23]. The gRNAs targeting the pig *ApoE/LDLR* genes and Cas9 expression plasmids (ratio, 1:1) were co-transfected into PEFs of log-growth phase at a concentration of 5 μg/10^6^ cells. The transfection procedure was conducted on the platform of Nucleofector™ 2b Device (Lonza, Basel, Switzerland), according to the manufacturer's guidelines and using program T-016. Transcription efficiency was evaluated with EGFP plasmid (Supplementary Figure 2). The transfected cells were cultured at 37.0 °C for 48 hours in complete DMEM (Gibco) medium containing 20% FBS (Thermo Fisher Scientific, Shanghai, China) for recovery. The medium was replaced with complete DMEM medium containing 10% FBS and 0.5 μg/mL puromycin (Sigma-Aldrich, St. Louis, USA) after 24 hours for enrichment. The DNA of the cell pool was subjected to PCR amplification using primers for pig *ApoE* or *LDLR* (LDLR1), which are listed in Supplementary Table 2. The PCR products were applied to a T7EN1 cleavage assay and sub-cloned into the pMD18-T vector for TA cloning (TaKaRa, Code No. 6011). The T7EN1 cleavage assay was performed according to Shen et al. [24]. Bacterial TA colonies were randomly picked to perform sequencing.

PEF cell transfection and genotyping

The gRNA targeting pig *ApoE/LDLR* and Cas9 expression plasmids (1:1:2 for *ApoE/LDLR*) were co-transfected into PEFs as mentioned above. The cells were cultured in drug selection conditions for 4-5 days, followed by removal of the puromycin. Following approximately another 7 days of culture, the single cell colonies were transferred to a 48-well plate. When the cells reached full confluency, one half of each individual colony was lysed as previously described for PCR screening [19], and the remaining cells were subcultured for SCNT. The detailed information of these primers for pig *ApoE or LDLR* (LDLR2) are listed in Supplementary Table 2. The PCR products of all positive cell colonies were used for TA cloning and Sanger sequencing.

SCNT and embryo transfer

The manipulation of SCNT in the pigs was performed according to Lai and Whitworth [19, 25]. The detailed process is as follows. *In vitro* matured porcine oocytes were denuded with hyaluronidase at a concentration of 1 mg/mL. The matured oocytes that were in the MII stage of meiosis were placed in manipulation medium containing 7 μg/mL of cytochalasin B. They were then enucleated and injected into transgenic cells into the same position under the zona pellucida. After 15 mins of equilibration in manipulation medium, the electrical fusion and activation process followed. Reconstructed gene-edited embryos were cultured in PZM-3 medium for 12-24 h, and then the high quality cloned embryos were transplanted into surrogate sows. The pregnancy status of the sows was monitored by ultrasonic examination after 30 days. The cloned piglets were obtained by natural birth within the due date range of 114-120 days [25].

Genotype and off-target analysis gene-targeted pigs

The genome of all six founder pigs were collected from ear tissues for genotype analysis. The primers for the *ApoE*/*LDLR* genes as well as PCR reaction conditions were mentioned above. The PCR products were applied for TA cloning.

The rule of OTs prediction in the pig genome is noted in previous reports [10, 12, 23]. The putative off-target sites were predicted using CCTop; the parameter of maximum total mismatches was set at 5, the core length was 12 nt, the maximum core mismatch was set to 2 nt, and PAM was set as NRG (= NGG or NAG) [26]. The DNA fragments covering the off-target site were amplified via PCR using the genomic DNAs extracted from the founder ear tissues and the wild-type PEF cell line. The sites that were successfully amplified were subjected to Sanger sequencing and the TA clone assay. Supplementary Table 3 summarizes all the primers corresponding to the off-target sites.

Serum biochemical analyses

Blood samples were drawn from precaval veins into a serum separation hose (BD Vacutainer SST Tubes, Shanghai, China) after an overnight fast. The TC, TG, HDL-C and LDL-C were measured by the clinic laboratory in the 309th Hospital of Chinese People's Liberation Army (Beijing, China). The serum APOB was determined with the porcine APOB ELISA kit (Lembo Terry Technology Development, Beijing, China). The serum ApoE was determined with the porcine ApoE ELISA kit (Lembo Terry Technology Development, Beijing).

Statistics

The experimental data were analyzed using Prism software (GraphPad Software). Multiple comparisons of data from multiple groups were performed using analysis of variance (ANOVA). The data are presented as numerical means ± SEM. The level of significance is P<0.05.

ACKNOWLEDGMENTS

This work was supported by grant from the Major National Scientific Research Projects (2015CB943101), National Science and Technology Major Project (2016ZX08010004), International Advanced Agricultural Science and Technology Import Program (2016-X36) and the Agricultural Science and Technology Innovation Program (ASTIP-IAS05) of CAAS.

CONFLICTS OF INTEREST

No competing financial interests exist.

REFERENCES

1. Gutierrez LL, Maslinkiewicz A, Curi R, de Bittencourt PI Jr. Atherosclerosis: a redox-sensitive lipid imbalance suppressible by cyclopentenone prostaglandins. Biochem Pharmacol. 2008; 75:2245-62. doi: 10.1016/j.bcp.2008.03.002.

2. Georgiadou D, Chroni A, Vezeridis A, Zannis VI, Stratikos E. Biophysical analysis of apolipoprotein E3 variants linked with development of type III hyperlipoproteinemia. PLoS One. 2011; 6:e27037. doi: 10.1371/journal.pone.0027037.

3. Davis BT, Wang XJ, Rohret JA, Struzynski JT, Merricks EP, Bellinger DA, Rohret FA, Nichols TC, Rogers CS. Targeted disruption of LDLR causes hypercholesterolemia and atherosclerosis in Yucatan miniature pigs. PLoS One. 2014; 9:e93457. doi: 10.1371/journal.pone.0093457.

4. Langheinrich AC, Michniewicz A, Bohle RM, Ritman EL. Vasa vasorum neovascularization and lesion distribution among different vascular beds in ApoE-/-/LDL-/- double knockout mice. Atherosclerosis. 2007; 191:73-81. doi: 10.1016/j.atherosclerosis.2006.05.021.

5. Al-Mashhadi RH, Sorensen CB, Kragh PM, Christoffersen C, Mortensen MB, Tolbod LP, Thim T, Du Y, Li J, Liu Y, Moldt B, Schmidt M, Vajta G, et al. Familial hypercholesterolemia and atherosclerosis in cloned minipigs created by DNA transposition of a human PCSK9 gain-of-function mutant. Sci Transl Med. 2013; 5:166ra1. doi: 10.1126/scitranslmed.3004853.

6. Reyes LM, Estrada JL, Wang ZY, Blosser RJ, Smith RF, Sidner RA, Paris LL, Blankenship RL, Ray CN, Miner AC, Tector M, Tector AJ. Creating class I MHC-null pigs using guide RNA and the Cas9 endonuclease. J Immunol. 2014; 193:5751-7. doi: 10.4049/jimmunol.1402059.

7. Chen F, Wang Y, Yuan Y, Zhang W, Ren Z, Jin Y, Liu X, Xiong Q, Chen Q, Zhang M, Li X, Zhao L, Li Z, et al. Generation of B cell-deficient pigs by highly efficient CRISPR/Cas9-mediated gene targeting. J Genet Genomics. 2015; 42:437-44. doi: 10.1016/j.jgg.2015.05.002.

8. Vilahur G, Padro T, Badimon L. Atherosclerosis and thrombosis: insights from large animal models. J Biomed Biotechnol. 2011; 2011:907575. doi: 10.1155/2011/907575.

9. Zhou X, Wang L, Du Y, Xie F, Li L, Liu Y, Liu C, Wang S, Zhang S, Huang X, Wang Y, Wei H. Efficient Generation of Gene-Modified Pigs Harboring Precise Orthologous Human Mutation via CRISPR/Cas9-Induced Homology-Directed Repair in Zygotes. Hum Mutat. 2015. doi: 10.1002/humu.22913.

10. Wang K, Ouyang H, Xie Z, Yao C, Guo N, Li M, Jiao H, Pang D. Efficient Generation of Myostatin Mutations in Pigs Using the CRISPR/Cas9 System. Sci Rep. 2015; 5:16623. doi: 10.1038/srep16623.

11. Wang X, Zhou J, Cao C, Huang J, Hai T, Wang Y, Zheng Q, Zhang H, Qin G, Miao X, Wang H, Cao S, Zhou Q, et al. Efficient CRISPR/Cas9-mediated biallelic gene disruption and site-specific knockin after rapid selection of highly active sgRNAs in pigs. Sci Rep. 2015; 5:13348. doi: 10.1038/srep13348.

12. Zhou X, Xin J, Fan N, Zou Q, Huang J, Ouyang Z, Zhao Y, Zhao B, Liu Z, Lai S, Yi X, Guo L, Esteban MA, et al. Generation of CRISPR/Cas9-mediated gene-targeted pigs via somatic cell nuclear transfer. Cell Mol Life Sci. 2014. doi: 10.1007/s00018-014-1744-7.

13. Bordignon V, El-Beirouthi N, Gasperin BG, Albornoz MS, Martinez-Diaz MA, Schneider C, Laurin D, Zadworny D, Agellon LB. Production of cloned pigs with targeted attenuation of gene expression. PLoS One. 2013; 8:e64613. doi: 10.1371/journal.pone.0064613.

14. Wang Y, Du Y, Shen B, Zhou X, Li J, Liu Y, Wang J, Zhou J, Hu B, Kang N, Gao J, Yu L, Huang X, et al. Efficient generation of gene-modified pigs via injection of zygote with Cas9/sgRNA. Sci Rep. 2015; 5:8256. doi: 10.1038/srep08256.

15. Holm IE, Alstrup AK, Luo Y. Genetically Modified Pig Models for Neurodegenerative Disorders. J Pathol. 2015. doi: 10.1002/path.4654.

16. Carrington B, Varshney GK, Burgess SM, Sood R. CRISPR-STAT: an easy and reliable PCR-based method to evaluate target-specific sgRNA activity. Nucleic Acids Res. 2015. doi: 10.1093/nar/gkv802.

17. Wang X, Yu H, Lei A, Zhou J, Zeng W, Zhu H, Dong Z, Niu Y, Shi B, Cai B, Liu J, Huang S, Yan H, et al. Generation of gene-modified goats targeting MSTN and FGF5 via zygote injection of CRISPR/Cas9 system. Sci Rep. 2015; 5:13878. doi: 10.1038/srep13878.

18. Hai T, Teng F, Guo R, Li W, Zhou Q. One-step generation of knockout pigs by zygote injection of CRISPR/Cas system. Cell Res. 2014; 24:372-5. doi: 10.1038/cr.2014.11.

19. Whitworth KM, Lee K, Benne JA, Beaton BP, Spate LD, Murphy SL, Samuel MS, Mao J, O'Gorman C, Walters EM, Murphy CN, Driver J, Mileham A, et al. Use of the CRISPR/Cas9 system to produce genetically engineered pigs from in vitro-derived oocytes and embryos. Biol Reprod. 2014; 91:78. doi: 10.1095/biolreprod.114.121723.

20. Peng J, Wang Y, Jiang J, Zhou X, Song L, Wang L, Ding C, Qin J, Liu L, Wang W, Liu J, Huang X, Wei H, et al. Production of Human Albumin in Pigs Through CRISPR/Cas9-Mediated Knockin of Human cDNA into Swine Albumin Locus in the Zygotes. Sci Rep. 2015; 5:16705. doi: 10.1038/srep16705.

21. Li Y, Fuchimoto D, Sudo M, Haruta H, Lin QF, Takayama T, Morita S, Nochi T, Suzuki S, Sembon S, Nakai M, Kojima M, Iwamoto M, et al. Development of Human-Like Advanced Coronary Plaques in Low-Density Lipoprotein Receptor Knockout Pigs and Justification for Statin Treatment Before Formation of Atherosclerotic Plaques. J Am Heart Assoc. 2016; 5:e002779. doi: 10.1161/JAHA.115.002779.

22. Ogita M, Miyauchi K, Onishi A, Tsuboi S, Wada H, Konishi H, Naito R, Dohi T, Kasai T, Kojima Y, Schwartz RS, Daida H. Development of Accelerated Coronary Atherosclerosis Model Using Low Density Lipoprotein Receptor Knock-Out Swine with Balloon Injury. PLoS One. 2016; 11:e0163055. doi: 10.1371/journal.pone.0163055.

23. Ruan J, Li H, Xu K, Wu T, Wei J, Zhou R, Liu Z, Mu Y, Yang S, Ouyang H, Yanru Chen-Tsai R, Li K. Highly efficient CRISPR/Cas9-mediated transgene knockin at the H11 locus in pigs. Sci Rep. 2015; 5:14253. doi: 10.1038/srep14253.

24. Shen B, Zhang J, Wu H, Wang J, Ma K, Li Z, Zhang X, Zhang P, Huang X. Generation of gene-modified mice via Cas9/RNA-mediated gene targeting. Cell Res. 2013; 23:720-3. doi: 10.1038/cr.2013.46.

25. Lai L, Prather RS. Production of cloned pigs by using somatic cells as donors. Cloning Stem Cells. 2003; 5:233-41. doi: 10.1089/153623003772032754.

26. Stemmer M, Thumberger T, Del Sol Keyer M, Wittbrodt J, Mateo JL. CCTop: An Intuitive, Flexible and Reliable CRISPR/Cas9 Target Prediction Tool. PLoS One. 2015; 10:e0124633. doi: 10.1371/journal.pone.0124633.
